# Adjacent regenerative peripheral nerve interfaces produce phase-antagonist signals during voluntary walking in rats

**DOI:** 10.1186/s12984-017-0243-0

**Published:** 2017-04-24

**Authors:** Daniel Ursu, Andrej Nedic, Melanie Urbanchek, Paul Cederna, R. Brent Gillespie

**Affiliations:** 1 Department of Mechanical Engineering, University of Michigan, Ann Arbor, MI USA; 2 Department of Surgery, Plastic Surgery Section, University of Michigan Health System, Ann Arbor, MI USA

**Keywords:** Peripheral nerve interface, Prosthetics, Regenerative medicine, Amputees

## Abstract

**Background:**

Regenerative Peripheral Nerve Interfaces (RPNIs) are neurotized muscle grafts intended to produce electromyographic signals suitable for motorized prosthesis control. Two RPNIs producing independent agonist/antagonist signals are required for each control axis; however, it is unknown whether signals from adjacent RPNIs are independent. The purpose of this work was to determine signaling characteristics from two adjacent RPNIs, the first neurotized by a foot dorsi-flexor nerve and the second neurotized by a foot plantar-flexor nerve in a rodent model.

**Methods:**

Two Control group rats had electrodes implanted onto the soleus (tibial nerve) and extensor digitorum longus (peroneal nerve) muscles in the left hind limb. Two Dual-RPNI group rats had two separate muscles grafted to the left thigh and each implanted with electrodes: the extensor digitorum longus was neurotized with a transected fascicle from the tibial nerve, and the tibialis anterior was implanted with a transected peroneal nerve. Four months post-surgery, rats walked on a treadmill, were videographed, and electromyographic signals were recorded. Amplitude and periodicity of all signals relative to gait period were quantified. To facilitate comparisons across groups, electromyographic signals were expressed as a percent of total stepping cycle activity for each stance and swing gait phase. Independence between peroneal and tibial nerve activations were assessed by statistical comparisons between groups during stance and swing.

**Results:**

Electromyographic activity for Control and Dual-RPNI rats displayed alternating activation patterns coinciding with stance and swing. Significant signal amplitude differences between the peroneal and tibial nerves were found in both the Control and Dual-RPNI groups. Non-inferiority tests performed on Dual-RPNI group signal confidence intervals showed that activation was equivalent to the Control group in all but the peroneal RPNI construct during stance. The similar electromyographic activity obtained for Control and RPNI suggests the latter constructs activate independently during both stance and swing, and contain minimal crosstalk.

**Conclusions:**

In-vivo myoelectric RPNI activity encodes neural activation patterns associated with gait. Adjacent RPNIs neurotized with agonist/antagonist nerves display activity amplitudes similar to Control during voluntary walking. The distinct and expected activation patterns indicate the RPNI may provide independent signaling in humans, suitable for motorized prosthesis control.

## Background

Acquiring a sufficient number of independent peripheral nerve signals from an amputee’s residual limb is critical to the control of an advanced prosthetic device. Despite the existence of sophisticated multiple degree of freedom (DOF) prosthetic devices, demonstration of smooth, multifunctional control has been limited and the search continues for an optimal interface between human and prosthesis [1]. Targeted muscle reinnervation (TMR), which employs nerve transfers to reinnervate specific muscle sites, is the most immediately applicable interfacing strategy that has been demonstrated to provide neural input signals for prosthetic control [2]. Other strategies include direct brain interfaces, which have also been successfully tested in humans, but are generally considered too invasive and high-risk for the limb loss population [3].

In parallel, peripheral nerve interfaces have been extensively studied using cuff electrodes placed on whole peripheral nerves [4, 5], cuff electrodes spanning individual nerve fascicles [6, 7], and intra-axonal electrodes [8]. Peripheral neurography (pNG) signals suitable for prosthetic control have been recorded using longitudinal intrafascicular electrodes [9]. By bridging such electrodes to distinct motor and sensory neuro-fascicular bundles, both graded movement of and sensation from a prosthetic device have been demonstrated [10, 11]. Even finer sensory perception (up to 81 palmar locations) and motor control (up to 13 distinct finger movements) has been achieved with offline decoding of signals from a 100-electrode Utah Slanted Electrode Array placed on the ulnar nerve of an amputee [12]. However, mechanically induced neural injury at the electrode-nerve interface and bio-compatibility complications involving scar formation on neural tissue [13–15], are ongoing considerations that limit long term use [16, 17].

An alternative interface currently under development is the Regenerative Peripheral Nerve Interface (RPNI), which uses a muscle graft to connect between a severed nerve and the electronics of a prosthetic device [18]. Specifically, an RPNI device consists of a nonvascularized 300-600 milligram skeletal muscle graft that is implanted with, and subsequently neurotized by a transected peripheral nerve. Through the muscle graft, nerve signals can be transduced, amplified, and detected by either epimysial or intramuscular electrodes [19]. Unlike TMR, the RPNI is not restricted to the utilization of vascularized muscle within the residual limb or the nearby chest wall, thereby permitting physiologically relevant connections to individually functioning fascicles within the peripheral nerve. The small size of the RPNI holds promise for the placement of multiple such constructs in a confined space such as the forearm. Furthermore, by connecting the severed nerve to a muscle graft, the RPNI device also prevents neuroma formation.

Previous studies performed in anesthetized rats have shown the feasibility and durability of the RPNI construct [19–25]. Investigation of RPNI function in awake, walking rats demonstrated that in vivo myoelectric RPNI activity is periodic and entrained with gait, with signal amplitudes similar to controls, and minimal signal contamination from muscles adjacent to the RPNI [26]. While this study demonstrated the viability of the RPNI as a transducer for signals on peripheral nerves during rodent walking, it did not assess the performance of multiple adjacent but antagonistic RPNIs.

This paper examines the potential for creating multiple adjacent RPNIs in the same limb, with the signals of each construct encoding a different function. The RPNI is created with fascicles of peripheral nerves known to function as antagonist pairs during voluntary hind limb locomotion. RPNI signals are acquired from 2 rats, and compared to signals obtained from similarly functioning muscles of 2 Control rats during treadmill walking. Our main performance metrics for RPNI characterization are signal amplitude and signal correlation with hind limb joint kinematics. We expect antagonist pair signal activation from the RPNIs to be similar in amplitude and function to Controls.

## Methods

Four male 3-month-old F344 strain rats were used in this study. All animal care and use procedures were conducted in accordance with the National Research Council’s *Guide for the Care and Use of Laboratory Animals* (1996) and were approved by the University of Michigan Animal Care and Use Committee.

### Surgical preparation

**Fig. 1:**
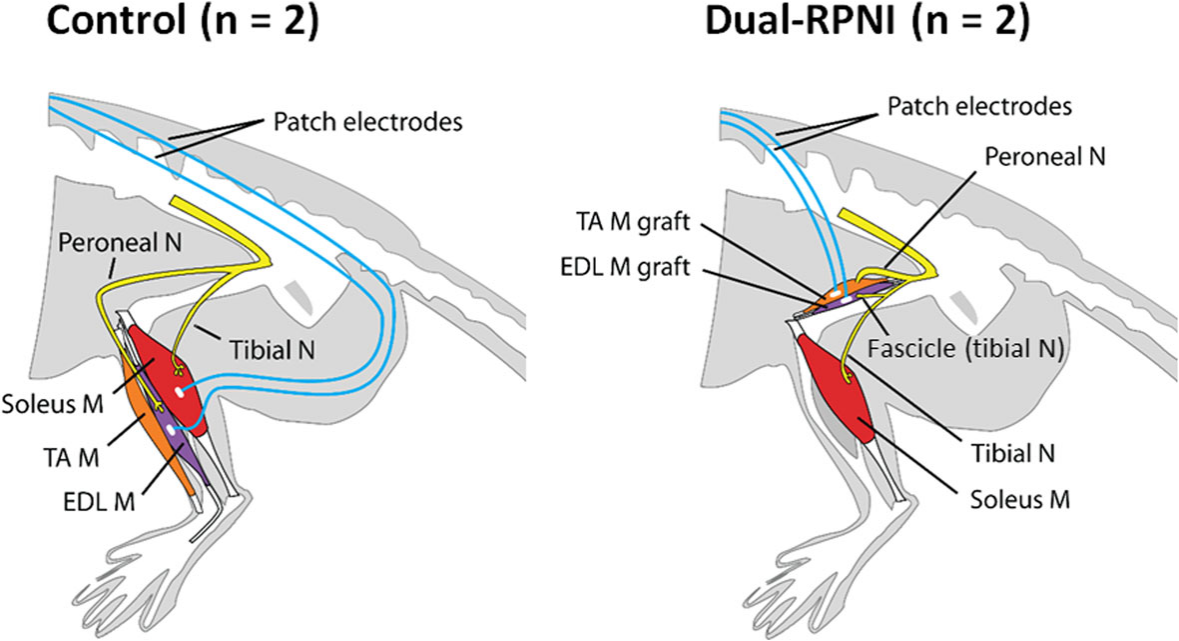
Surgical preparation of Control and Dual-RPNI rats. Schematic Diagram of the left hind limbs, indicating nerves, muscles, and bipolar electrode placement for the Control and Dual-RPNI study groups. The only surgical intervention for rats in the Control group involved electrode placement. Rats in the Dual-RPNI group underwent a free EDL muscle transfer with placement of a fascicle of the tibial nerve, and a free tibialis anterior muscle transfer with placement of the peroneal nerve. Both muscle transfers were placed adjacent to one another, and anchored to the femur in the left hind limb. Abbreviations: M = muscle; N = nerve

**Fig. 2:**
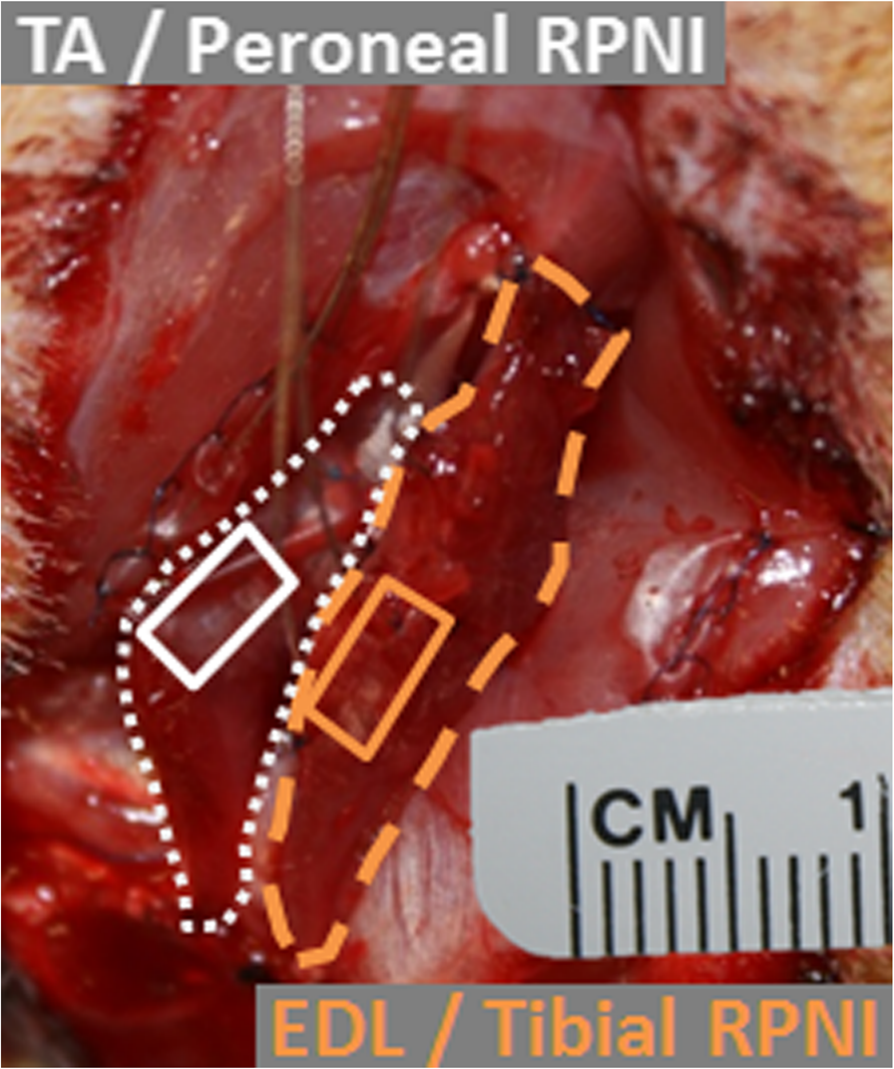
Intra-operative photo of the left hind limb of a Dual-RPNI rat, indicating TA (*dotted region*) and EDL (*dashed region*) muscle graft locations, and bipolar electrode placement on each muscle graft epimysium (*solid region*). For the purpose of minimizing signal cross-talk, electrodes were placed on opposite sides of each muscle graft

Two Control group rats, in which the neuromuscular anatomy remained intact, received two bipolar patch electrodes, one on the left soleus and another on the extensor digitorum longus (EDL) muscles. Two Dual-RPNI group rats received a free left tibialis anterior (TA) muscle transfer, and a free left EDL muscle transfer to the left thigh. Both muscles were sutured adjacent to the femur. The TA was neurotized with the proximal end of the transected peroneal nerve, while the EDL was neurotized with a fascicle of the tibial nerve, transected from one head of the gastrocnemius muscle. The remainder of the tibial nerve was left intact, so as to provide innervation to the posterior compartment of the lower hind limb. Each muscle was equipped with a bipolar patch electrode (Fig. 1). To minimize signal cross-talk between muscle grafts, the patch electrode on the EDL muscle graft was placed on top of the muscle belly just below the skin, while the electrode on the TA muscle graft was placed inferior to the muscle, abutting the femur (Fig. 2). In both groups, the bipolar patch electrodes (Double Standard, EP203 Customized; Microprobes, Gaithersburg, MD) were sutured to the respective muscles epimysium. The patch consisted of Teflon^®;^ insulated fine stranded stainless steel leads embedded in Dacron^®;^ reinforced silicone rubber, 0.18 mm thick. A piece of decellularized small intestinal submucosa (Surgisis, Cook Biotech, West Lafayette, IN) was trimmed to size, hydrated, sterilized with 70% alcohol, rinsed, and then wrapped around each muscle-electrode unit to secure the electrodes in place. The cables from the electrodes were tunneled subcutaneously and secured to a head cap that was fixed to the skull using cortical screws and methyl methacrylate (Sigma-Aldrich, Co. LLC, St. Louis, MO). The surgical sites were then closed with suture.

Each rat recuperated for 4 months to facilitate wound healing and, for the Dual-RPNI group, reinnervation of the free muscle grafts. On the day of an evaluation, each rat’s left hind limb was shaved; on the bare skin, non-toxic clay based paints were used to mark the positions of the hip, knee, and ankle joints, as well as the distal end of the paw. Wire electrode ends were accessed through the head cap and connected to the electromyographic (EMG) recording apparatus.

### Recording apparatus

Rats were conditioned to walk on a rat treadmill (Columbus Instruments, Columbus OH) at constant pace between 8.5 and 9.0 m/min. One high speed camera capable of recording at 120 frames per second (GoPro Hero2, San Mateo, CA) was positioned with its field of view perpendicular to the rat’s walking direction and activated through a digital trigger. Myoelectric signals were amplified and bandpass filtered (1–500 Hz) with the use of a custom-built analog bipolar instrumentation amplifier. A nominal gain of 1000x was used and signal amplitudes were calibrated using a function generator and oscilloscope. The amplified and filtered signal was acquired at a 3 kHz sampling rate; during post-processing, it was digitally rectified and zero-phase low-pass filtered to 50 Hz. An LED positioned within the field of the camera was toggled off and on through a button press. The LED control signal was recorded by the data acquisition system to facilitate synchronization between the video and myoelectric recordings.

A custom video and data processing program was written in MATLAB (Mathworks, Natick, MA) to facilitate synchronization of the video and myoelectric recordings and extraction of hind limb kinematics from the video. The LED appearing in the video enabled the motion recordings to be synchronized to the LED control signal recorded alongside the myoelectric signals. Within the video processing program, a computer vision algorithm was written in MATLAB to extract centroid locations for each of the left hip, knee, ankle, and toe color markers appearing in each frame of the video recordings. These centroid locations were used in turn to identify hip, knee, ankle and toe joint angle trajectories of the left hind limb.

The identified centroids and limb segments were displayed in overlay on the video recordings. The visualization program’s user interface supported the addition of gait event markers to the dataset. Gait events indicating left paw landing and lift-off were added to each dataset, allowing the categorization of the gait data into periods of stance (landing to lift-off) and swing (lift-off to landing).

The extracted kinematic and EMG data was divided into bouts, wherein each bout contained data for four or more complete strides. Walking was defined as a bout during which one or the other hind limbs was in contact with the treadmill belt. Sequences in which the rat was standing still or hopping were removed. These data were used to assess instantaneous EMG signal strength during walking cycles, and assess the cross-correlation between ankle joint kinematics and EMG data during stance and swing phases of gait.

### Data analysis

To assess EMG signal strength from all groups, the area under the curve of the filtered EMG data collected during each step of the walking bouts was calculated. These data were then segmented into stance and swing and normalized across the gait period, such that signals acquired for one gait during stance and swing summed to 100%.

Two statistical tests were performed in order to first, differentiate between the EMG activity transduced from different nerve signals within the same study group, and second, assess whether EMG activity transduced from the same nerve type across the two study groups can be considered equivalent. In order to statistically differentiate between the EMG signal activity transduced from each nerve within a study group, paired two-sample t-Test comparisons were performed between the signals transduced from the peroneal and tibial nerves within the Control and Dual-RPNI groups, respectively. Equivalence between the EMG signal activity transduced from the Control and Dual-RPNI peroneal nerves, and the Control and Dual-RPNI tibial nerve innervated musculature was assessed using the statistical non-inferiority test [27], using the bio-equivalence guidelines proposed by the United States Food and Drug Administration [28]. All statistical computations were performed using SPSS Statistics 22, (SPSS, IBM Inc., 2013, Armonk, NY). Significance levels were set to *α*=0.05.

In detail, the EMG signals transduced from the tibial nerve of the Control group during stance (or swing) were used to calculate the EMG percent activity mean, *μ*_*ref*_, and standard deviation, *s*_*ref*_. Similarly, the EMG percent activity mean, *μ*_*test*_, and standard deviation, *s*_*test*_, was calculated for the signals transduced during stance from the tibial nerve of the Dual-RPNI group. A ±20*%* interval of equivalence was calculated around *μ*_*ref*_, and subtraction of *μ*_*ref*_ from this interval resulted in a window with lower bound *θ*_*L*_ and upper bound *θ*_*U*_. Next, a 100−*α* *%* (i.e. 95%) Confidence Interval was computed for the difference in means between *μ*_*test*_ and *μ*_*ref*_, such that the lower bound *c*_*L*_ and upper bound *c*_*U*_ of this interval is given by the formula:

1 $$\begin{array}{*{20}l}  [\!c_{L},c_{U}]=\mu_{test}-\mu_{ref}\mp 1.96\sqrt{\frac{s_{test}^{2}}{n_{test}}+\frac{s_{ref}^{2}}{n_{ref}}} \end{array} $$

where *n* is the number of samples (32) and 1.96 is the Z-score value at *z*_(1−*α*/2)_. Using this method, a conclusion of equivalence is supported with 95% probability if [*c*_*L*_,*c*_*U*_] is contained in the interval [*θ*_*L*_,*θ*_*U*_]. The same procedure was repeated separately for comparing Control and Dual-RPNI peroneal nerve signals during stance and swing, respectively.

For the purpose of comparing kinematic and EMG data between groups for an average step, left hind paw gait events were used to segment the kinematic and EMG recordings into stance and swing phases of gait. Stance was defined between the left hind paw making contact with the treadmill (landing) and subsequent liftoff. Swing was defined between liftoff and the next landing event. Each gait cycle (including kinematic and myoelectric recordings) was time-course normalized such that the beginning of stance corresponded to 0% and end of swing corresponded to 100% of gait for the left hind limb. Within each experimental group, normalized kinematic and myoelectric recordings were aligned using the landing gait events before being used to compute a mean and standard deviation representative of a single step for all left hind limb joint angles and the acquired EMG data.

## Results

**Fig. 3:**
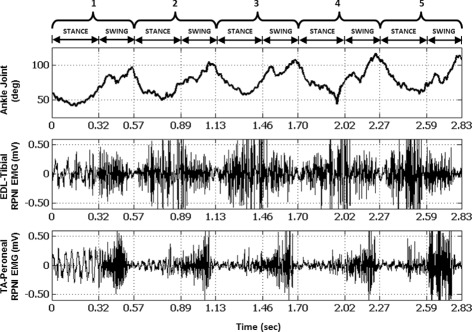
Raw kinematic and myoelectric data obtained from Control and Dual-RPNI rats. Ankle kinematic (*top*) and myoelectric data (*center*, *bottom*) obtained from the left hind limb of a Dual-RPNI rat, equipped with two adjacently placed RPNI interfaces during five consecutive steps of a walking task. The time periods are labeled according to whether the hind limb was in the stance or swing phase

Raw EMG signals were more active with higher amplitudes during walking sequences than during periods of standing still in both Control and Dual-RPNI rats. EMG signals from both of the Control and both of the Dual-RPNI rats exhibited an alternating pattern of activity during continuous walking. Figure 3 shows representative ankle joint kinematics and raw EMG activity from the two adjacent constructs in an RPNI rat during five consecutive steps. The time axis is labeled according to whether the hind limb was in the stance or swing phase. In particular, the signals recorded from both RPNIs during walking featured large excursions with peak to peak EMG voltage amplitudes ranging between 0.75 to 1.0 mV_*PP*_. These EMG voltage peaks occurred during different points in the gait cycle for each RPNI.

**Fig. 4:**
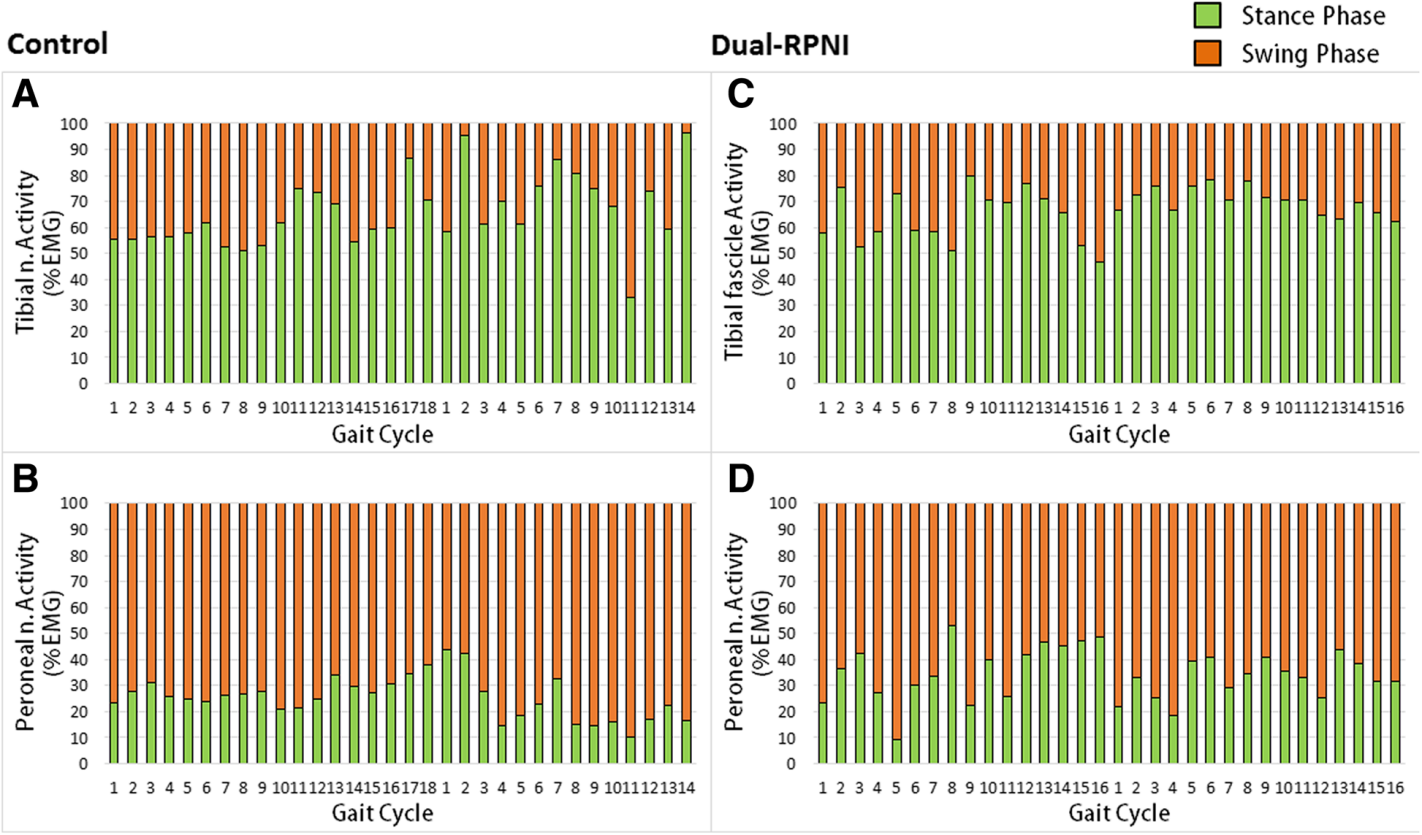
Percent EMG activity of Control and Dual-RPNI rat strides. EMG signal activity, illustrated as a percentage of the total gait cycle obtained from the musculature of 2 Control group rats (*left*) and 2 Dual-RPNI group rats (*right*) during 32 strides. Activity transduced from (**a**) tibial nerve (Control), (**b**) peroneal nerve (Control), (**c**) tibial nerve (Dual-RPNI) and (**d**) peroneal nerve (Dual-RPNI) has been segmented into stance and swing for each step

Walking EMG signal strength for each experimental group was quantified by integration of the area under the curve of the filtered EMG traces obtained per stride. Figure 4 illustrates the myoelectric activity (depicted as percent activity during stride) transduced from the tibial (*top*) and peroneal nerve (bottom) signals of the Control (*left*) and Dual-RPNI rats (*right*) during 32 walking steps. Comparison between the signals transduced from similar nerves shows matching activity profiles in Control and Dual-RPNI rats during stance and swing.

**Fig. 5:**
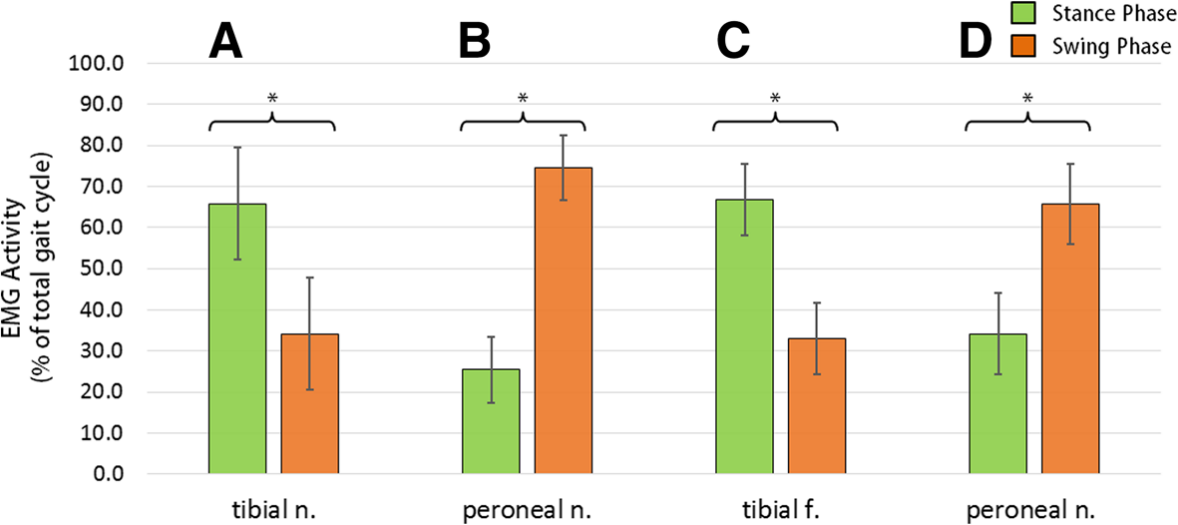
Mean and standard deviation of Control and Dual-RPNI rat EMG signal activity. Mean ± one standard deviation of EMG signal activity obtained during stance and swing phases of gait, illustrated as a percentage of the total gait cycle obtained from (**a**) tibial nerve (Control), (**b**) peroneal nerve (Control), (**c**) tibial nerve (Dual-RPNI) and (**d**) peroneal nerve (Dual-RPNI). Within group t-test comparisons between stance and swing phase percent EMG activity are significant with (*p*<0.05)

Paired two-sample t-Test comparisons were made to statistically differentiate between the mean EMG signal activity obtained from Control rats. A similar comparison was performed for the Dual-RPNI EMG data. Figure 5 presents the mean and standard deviation of the EMG activity obtained from the Control and Dual-RPNI group rats during walking. Within-group comparisons of Control and Dual-RPNI signals demonstrate a significant difference (*p*<0.05) between the EMG activity transduced from the tibial and peroneal nerves in either group.

Non-inferiority tests for equivalence of Dual-RPNI EMG signals obtained from muscles innervated with the same nerve as Controls were performed using a 20% equivalence window centered around the corresponding mean Control EMG activity. Equivalence of Dual-RPNI signal activity was supported if the difference in means 95% Confidence Interval (calculated using Eq. 1) was contained within the 20% equivalence window devised around the Control mean. The equivalence test results performed between the Dual-RPNI and Control signals obtained during stance and swing for each nerve type are presented in Table 1. Using this criterion, all acquired Control and Dual-RPNI signals were found to be significantly equivalent, (*p*<0.05), except for EMG activity transduced from the peroneal nerve during stance.

**Table 1:** Statistical non-inferiority (equivalence) test results for comparisons made between Control and Dual-RPNI study groups using percent EMG activity calculated from 32 strides

	Phase	Study group	Mean	S D.	20% Window	95% C.I.	Non-inferior?	
			(*μ*)	(*s*)	[*θ*_*L*_,*θ*_*U*_]	[*c*_*L*_,*c*_*U*_]	[*c*_*L*_,*c*_*U*_]⊂[*θ*_*L*_,*θ*_*U*_]	
Tibial n.	Stance	Control	65.77	13	66	[-13.16, 13.16]	[ -4.49, 6.73 ]	Yes
		Dual-RPNI	66.89	8	68			
	Swing	Control	34.22	13	61	[ -6.84, 6.84 ]	[ -6.70, 4.47 ]	Yes
		Dual-RPNI	33.11	8	64			
Peroneal n.	Stance	Control	25.41	7	98	[-5.08, 5.08]	[4.42, 13.19]	No^a^
		Dual-RPNI	34.20	9	82			
	Swing	Control	74.58	7	96	[-14.91, 14.91]	[-13.17, -4.43]	Yes
		Dual-RPNI	65.78	9	79			

Comparisons between groups were made between signals acquired from similarly-innervated muscle as a function of gait phase. The 20% Window was calculated around each control mean. The 95% C.I. was calculated using Eq. 1. Statistical equivalence between respective Dual-RPNI and Control percent EMG activity is inferred with (*p*<0.05) if the 95% C.I. is completely contained within the 20% Window

^a^The non-inferiority criterion for Dual-RPNI percent EMG activity calculated from the peroneal innervated muscle graft during stance is met if the low signals acquired from the last seven gaits of the Control group peroneal nerve activity during stance (see Fig. 4 b) are considered outliers

Abbreviations: *N* nerve, *SD* standard deviation, *CI* confidence interval

**Fig. 6:**
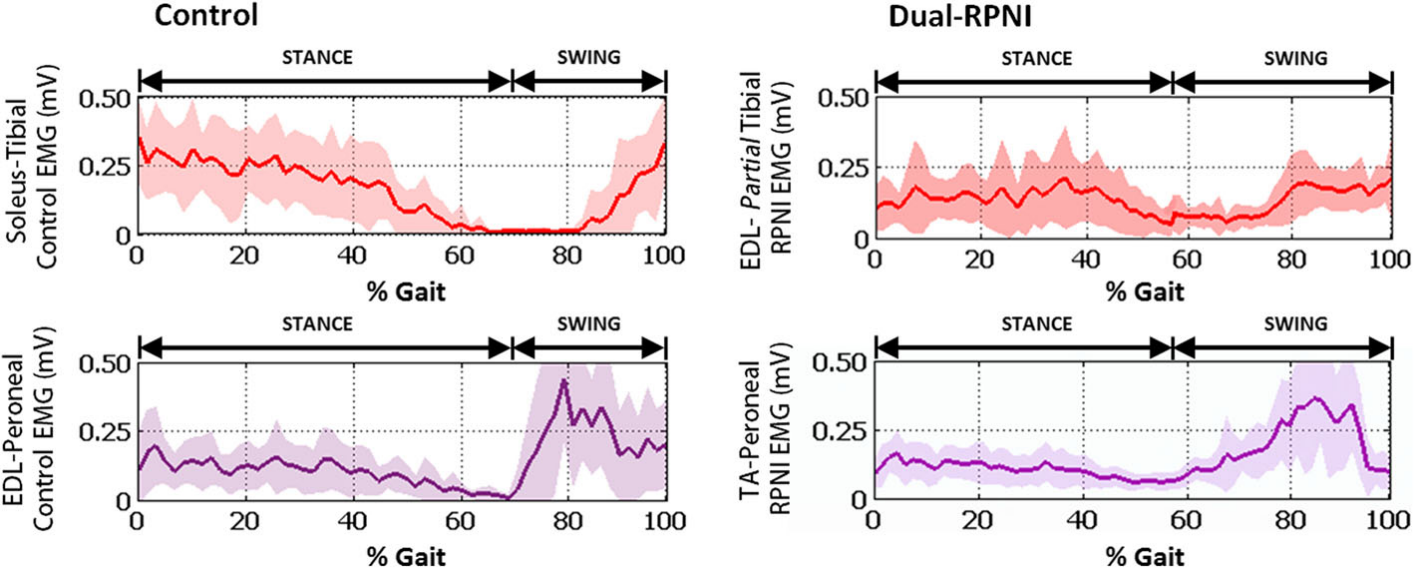
Time course average and standard deviation of Control and Dual-RPNI rat EMG and ankle joint kinematics. Time course average and standard deviation of 32 EMG signals, obtained from the musculature innervated with the tibial nerve (*red*) and peroneal nerve (*purple*) of 2 Control rats (*left*) and 2 Dual-RPNI rats (*right*) during walking. Traces have been normalized with respect to gait cycle, and separated into the stance and swing phases of gait

The EMG signal patterns extracted from the video recordings during 32 strides from the 2 Control and 2 Dual-RPNI rats were normalized in time to align foot landing events. Within study groups, these signals differed as a function of the nerve from which they originated. Between groups, similar nerves produced comparable EMG traces. In particular, the EMG signal transduced from the tibial nerve in both groups demonstrated more activity during the stance phase, when hind limb plantar-flexion is most pronounced. Peroneal nerve activity was increased during swing, when paw liftoff occurs via dorsi-flexion. Figure 6 depicts the time course averaged EMG signal obtained from the musculature innervated with the tibial nerve (red) and peroneal nerve (purple) of Control rats (left) and Dual-RPNI rats (right). Shaded regions behind the average trace indicate one standard deviation.

## Discussion

This exploratory study addresses important questions about the potential for using RPNIs in humans to acquire signals for voluntary control of a high DOF motorized prosthesis. Achieving *intuitive* prosthetic control requires that for each DOF, antagonist-pair RPNIs transduce signals in a manner that mimics the muscle activity of antagonist-pair muscles in the natural limb. Our previous work with single RPNIs placed bilaterally indicated that signals from RPNIs consistently exhibit signals similar in amplitude and pattern to myoelectric signals acquired from analogous muscles in control rats [26]. Furthermore, RPNI function was not corrupted by the presence of motion artifact or signals from neighboring muscles that contracted simultaneously, and was highly correlated with a walking task. These findings were promising, as RPNI constructs must be placed in the vicinity of other muscles, whose presence must not affect the RPNI output.

However, in that previous study, the use of whole peripheral nerves to create bilateral RPNIs in the rat hind limbs resulted in a marked gait alteration, and may explain the significantly different EMG activity profile demonstrated by the RPNIs, especially in the tibial nerve construct whose function during gait stance plantar-flexion is essential. To address this finding, and further characterize RPNI function, the study reported here has four new features: this study (1) employed RPNI constructs reinnervated with nerves known to exhibit antagonist activation, (2) placed RPNI constructs in proximity to one another, (3) used a fascicle (instead of the whole) tibial nerve to create an RPNI, and (4) harvested muscle grafts associated with different peripheral nerves than the nerves used to reinnervate the RPNIs.

In light of features (1) and (2), the present study demonstrated that RPNIs are capable of transducing the same out-of-phase neural signals as antagonist muscles performing mechanical work about a joint (see percent EMG activity as a function of gait phase in Fig. 5 and time course normalized traces in Fig. 6). The similarity of the activity profiles of these signals to controls demonstrates that adjacently placed RPNIs are not affected by crosstalk between constructs. Specifically, the peroneal nerve RPNI construct exhibited periods of signal activation and quiescence similar to Control signals obtained from the peroneal innervated EDL muscle, i.e. activation occurred during hind limb swing with relative quiescence exhibited during stance. The same can be noted of the tibial fascicle RPNI construct, when compared with signals obtained from the soleus. By contrast, the existence of significant crosstalk between the adjacent RPNIs would have affected the period of quiescence and antagonist pair activation of the two muscle grafts. This finding corroborates the observation from our previous study that RPNIs are not sensitive to noise emanating from adjacent contracting musculature.

Within a peripheral nerve, individual nerve fibers are grouped together in fascicles [29], and surgical procedures describing the dissection of peripheral nerves into distinct fascicular bundles are well established [30]. Clinical investigations (especially neurophysiological studeis) have also demonstrated that somatotopic clustering of nerve fibers within the fascicular bundle persists from the distal to the proximal end of the nerve [31]. This is in accord with the somatotopic organization known to exist for motor and sensory pathways in the CNS. In light of study feature (3), our approach showed that use of a nerve fascicle to reinnervate a muscle graft of different innervation origin provides profiles of signal activation above baseline similar to native muscle anatomically innervated by that same nerve. The signals in the RPNI constructs were not mirror images of their Control counterparts, which may be due to either selective or partial reinnervation of each muscle graft. This question will require further investigation. However, the existence of recognizable signal patterns suggests that certain fascicles of peripheral nerves can be used to create RPNIs with specific functions, while other fascicles are left in place to maintain innervation of existing musculature. This fascicular dissection was particularly valuable in the present study, as it resulted in the preservation of gait in a rat hind limb model. Moreover, in light of study feature (4), results support the idea that RPNIs can be created from multiple types of skeletal muscle tissue with no loss of overall signal information, as it relates to limb motion. This is particularly meaningful since reconstructive surgical procedures frequently make use of muscle grafts harvested from different parts of the body, where their function is either redundant or not essential for most activities of daily living. In such cases, each DOF of a prosthesis could be controlled by an antagonist-pair RPNI using a simple algorithm that compares the relative signal activity from each construct, without need for prior training or noise cancellation.

By employing a model of percent EMG signal activity during each stride of a walking task, this work demonstrated that adjacent RPNIs neurotized with agonist/antagonist nerves activate independently during voluntary walking, with signal activity patterns similar to Controls. Moreover, RPNIs constructed with fascicles rather than whole nerves and isografts of any musculo-motor origin produce signals in vivo similar to Controls. Together with previous longitudinal studies that investigated RPNI function in-situ, the present evaluation of RPNIs reinnervated with fascicles shows promise towards the creation of multiple construct antagonist pairs, each with a well-defined function providing a unique signal to one DOF in a computerized prosthetic device.

Certainly, a successful prosthetic interface should also incorporate sensory feedback, preferably by engaging the original neural circuits available in the limb. Investigations using intrafascicular electrodes have demonstrated that electrical stimuli can reestablish sensations of pressure and proprioception [10, 32–34]. The viability of using RPNI constructs to also provide sensory feedback and thereby close the sensori-motor loop in human users of an instrumented prosthesis will be explored in ongoing and future work.

## Conclusion

This exploratory study demonstrated that adjacently placed RPNIs reinnervated with nerves exhibiting antagonist-pair function provide out-of-phase trajectories similar in amplitude and activity to Controls. Placement of RPNI constructs in proximity to one another does not lead to crosstalk. Use of peripheral nerve fascicles instead of whole nerve does not alter RPNI function, and use of fascicles to reinnervate RPNI constructs may provide the advantage of transducing multiple independent signals for increased DOF control in a prosthesis. Moreover, muscle isografts of different innervation origin may be used to construct an RPNI, with no negative effects on its function. These findings suggest that adjacently paired RPNI control sites may yield improved multi-DOF fidelity for upper limb amputees utilizing a motorized prosthetic device.

## Abbreviations

CNS: Central nervous system
DOF: Degree of freedom
EDL: Extensor digitorum longus muscle
EMG: Electromyography
pNG: Peripheral neurography
RPNI: Regenerative peripheral nerve interface
TA: tibialis anterior muscle
TMR: Targeted muscle reinnervation
